# Editorial: Advances in the Pathophysiology, Diagnosis, and Treatment of Dry Eye Disease

**DOI:** 10.3389/fmed.2022.925876

**Published:** 2022-05-19

**Authors:** Yiteng Lu, Yuqing Wu, Xujiao Zhou, Takenori Inomata, Lei Gu, Xiuming Jin, Jiaxu Hong

**Affiliations:** ^1^Department of Ophthalmology, Eye and ENT Hospital, Fudan University, Shanghai, China; ^2^Department of Ophthalmology, Juntendo University Graduate School of Medicine, Tokyo, Japan; ^3^Epigenetics Laboratory, Max Planck Institute for Heart and Lung Research, Bad Nauheim, Germany; ^4^Eye Center, School of Medicine, Affiliated Second Hospital, Zhejiang University, Hangzhou, China

**Keywords:** dry eye, pathophysiology, diagnosis, treatment, risk factors, pathogenesis

## Introduction

Dry eye disease (DED) is a multifactorial disease of the ocular surface characterized by disruption of tear film homeostasis, causing ocular symptoms that include visual disturbance, discomfort, dryness and irritation of the eyes. In the development of DED, inflammation, tear hyperosmolarity and aberrant neurosensory functions are critical components which interact with one another to form a vicious cycle, leading to a chronic and progressive course of disease (1). The continually rising prevalence rate of DED, which affects millions of people in the United States and around the world, has become a major challenge for ophthalmologists (2). Yang W. et al. evaluated data from three eye facilities in a study of the economic impact of DED in China, finding that a patient's annual DED expenses can exceed $422.6, or to $166.6 billion per year nationally. The rising frequency of DED, as well as the immense economic burden it imposes, necessitates a greater understanding of its underlying pathophysiology and the development of effective diagnostic and therapeutic measures. As a result, we introduced the Research Topic “Advances in the Pathophysiology, Diagnosis, And Treatment of Dry Eye Disease” with the hopes of offering a comprehensive summary of the most recent findings on DED.

## Pathophysiology

### Risk Factors

As a multifactorial disease, the onset of DED is related to both intrinsic characteristics of the patient and external influences. Identification of these risk factors is crucial for research and early diagnosis of DED, yet the search for DED's risk factors is far from complete.

Several studies in this Research Topic have focused on the relationship between DED and other medical conditions which may be potential risk factors. Chen et al. conducted a study concerning children diagnosed with diabetes mellitus. At the end of their follow-up, 22.5% of the subjects developed DED, which is a prevalence rate much higher than that of normal children. Wu H. et al. explored that meibomian gland dysfunction (MGD) parameters were associated with the HbA1c level and diabetic duration and suggested that asymptomatic MGD may be an early indicator of DED onset. Gu et al. showed that SLE patients are at higher risk of developing DED which may result from poorer meibomian gland function. Chen et al. observed a tendency of schizophrenia patients developing DED signs and symptoms. A rise in inflammatory cytokines expression level was also detected within schizophrenia patients. However, this might be a result of antipsychotic medicine usage. Li S. et al. revealed that patients with severe obstructive sleep apnea hypopnea syndrome (OSAHS) exhibited worse conditions of upper lid meibomian gland and had higher incidence of DED compared with patients with non-severe OSAHS. They also pointed out that the damage of ocular surface cannot be reversed in a short time in OSAHS patients. Zheng et al. carried out a study including 257,932 patients to investigate the connection between DED and sleep disorders. The statistics showed a three-time increased risk for patients with sleep disorders to develop DED, which is a strong indicator of sleep disorders as a risk factor of DED based on the vast sample size. Consistently, Zhu Y. et al. reported that patients with poorer sleep qualities presented more extensive meibomian gland dropout, contributing to the development of evaporative DED. Hao et al. regarded Demodex folliculorum infestation as an important factor in the pathogenesis of DED based on higher prevalence of chalazion and worsen meibomian gland functions in infected patients.

Aside from infectious or non-modifiable medical conditions, the treatments patients receive, especially ophthalmic surgeries, are invasive and can irritate the ocular surface, therefore possibly giving rise to iatrogenic DED. Zhao S. et al. enrolled patients who underwent bilateral upper blepharoplasty and collected their DED associated parameters. They demonstrated that preexisting dry eye leads to a higher chance of persistent ocular surface damage along with higher levels of inflammatory cytokines. Similarly, Zhang S. et al. showed that blepharoplasty had a temporal negative effect on ocular surface and blinking functions. Chang et al. recruited 40 patients who underwent unilateral cataract surgery and found varying degrees of meibomian gland deterioration. Gong et al. evaluated the meibomian gland of patients subject to corneal refractive surgery and found a correlation between dry eye symptoms and preoperative meibomian gland status. Besides surgeries, the application of contact lens is another factor that may provoke the ocular surface given that they require lone-period contact and frequent change. Xu et al. reported that clinical signs and subclinical inflammation manifested at 1 week and 1 month post first-time contact lens wear respectively. The clinical signs may recover after quitting contact lens wear whereas inflammation tend to retain, suggesting a pathogenic effect of short term contact lens wear. In the long term, Yu H. et al. demonstrated that 2 years of orthokeratology lens wear increased lower eyelid meibomian gland dropout.

With the rapid development of technology, mobile phones and other smart devices have become an integral part of our daily lives. However, eye specialists are concerned since frequent use of smartphones and other electronic devices with screens is hazardous to the eyes and may increase the occurrence of DED. To clarify the relationship of DED and video display terminal (VDT) use, Zhao H. et al. discovered that increasing VDT use is associated with increased blinking, potentially leading to an increased risk of DED development, in which case a thicker lipid layer of the tear-film serves as a protective factor. Wang C. et al. investigated the influence of smartphone usage on the rate of dry eye diagnosis. They came to the conclusion that high-intensity smartphones use increases DED incidence, but that there is also an increase in false positive rate due to disturbed ocular surface homeostasis. Lin et al. investigated the immediate effects of VDT and found that following short-term usage of VDT, the tear-film lipid layer in patients with thick lipid layers decreased significantly. Notably, myopia has been linked to the smartphone usage (3). Wang N. et al. investigated the prevalence of DED in myopia patients in a cross-sectional study. The statistics revealed a 15.9% DED prevalence among 214 myopia patients, which was also correlated with eye rubbing and picky eating. In addition, many of the participants had various degrees of meibomian gland degeneration. Collectively, these findings add up to persuasive evidence that VDT exposure may be a significant risk factor for DED, either directly or indirectly.

### Pathogenesis

Among the complicated mechanisms of DED, inflammation is one of the most well-established factors for DED generation and has long been a hotspot for pathophysiology research. The role of long non-coding RNAs (lncRNAs) in DED was explored by Yang, Chen et al. After profiling the lncRNAs involved in DED, they identified two of the most significantly altered lncRNAs, Chrnb2 and Gabarapl2, for further research. Chrnb2 and Gabarapl2 silencing led to a down regulation of dry-eye related inflammatory cytokines including IL-6, TNF-α and IL-1β, suggesting that Chrnb2 and Gabarapl2 may play a role in inflammation regulation in DED. Alam et al. investigated the immunological response of a mouse strain with an RXRα mutation, which manifests dry eye-associated degenerative alterations with aging. They revealed that RXRα prevents γδT and conventional T cells from producing IL-17, halting the progression of DED. In DED models, Zhou et al. discussed the effect of WAY-100635, a 5-HT1A receptor antagonist, and found that WAY-100635 alleviated DED symptoms as well as inflammatory responses. Contrarily, 5-HT1A receptor agonist had the opposite effects. They also found that WAY-100635 had an effect on autophagy through ROS regulation. Wang B. et al. looked into the oxidative stress in DED and discovered that dual oxidase 2 (DUOX2), a ROS-producing enzyme, is elevated in DED. TLR4 was involved in the activation of DUOX2 and gave rise to oxidative stress, which in turn promoted HMGB1 release and inflammation. Morphologically, Zheng et al. evaluated patients with MGD with *in vivo* confocal microscopy (IVCM) and observed considerable amount of inflammatory cell infiltration in their meibomian glands.

In recent years, the relationship between microbiota and diseases has become an emerging topic (4). Song et al. studied the microbial communities and microbiome profiles of DED patients. They detected a reduced microbiome diversity in patients with DED compared with normal population. The composition of microbiome was similar in DED patients with or without SS, whereas the proportions were significantly different. By colonizing mice with microbiota from SS patients, Schaefer et al. confirmed the decreased microbiome diversity in their research and further highlighted the significance of the dysbiotic microbiome in the pathogenesis of DED. Interestingly, the transferred microbiota resulted in a decrease in regulatory T cells and ocular barrier function, which can be passed on to the offspring. Notably, the bacterial community on the ocular surface is altered by the injection of sodium hyaluronate, a medicine that is most widely used for DED therapy, regardless of preservative addition, according to Zhong et al.

On a global perspective, Yang et al. provided a comprehensive understanding of the ion-channels distributed on the ocular surface. Zhu, Inomata et al. summarized the animal models utilized for DED research. They classified and depicted each model with detailed description in terms of the underlying pathophysiologic mechanisms and measures of establishment. The thoroughly sorted information granted researchers a vast and more precise choice for animal models when studying DED.

## Diagnosis

The early and precise diagnosis of DED is beneficial to treatment and necessary for favorable prognosis. Unfortunately, DED shares a set of symptoms with a variety of other ocular surface diseases, making it difficult to distinguish between them. Wu Y. et al. systematically reviewed the current diagnostic measures in DED and summarized the most recent breakthroughs in novel examination approaches. They provided a valuable resource for DED diagnosis choices by assessing the indications and limitations of each procedure. Wang J. et al. designed an eyelid pressure measuring device for DED assessment. They found a correlation between eyelid pressure and ocular surface parameters. They also demonstrated that the device is safe and accurate. For examination of corneal sub-basal nerve plexus images derived from IVCM, Zhang Y. et al. compared a fully automated software, ACCMetrics, with a semi-automated one. ACCMetrics showed a reliable diagnostic performance. Similarly, Zhang Y-Y. et al. applied artificial intelligence for IVCM image processing, which also demonstrated excellent accuracy. These approaches, with further upgrade, might prove to be practical tools for DED diagnosis. For DED induced by systemic disease, Huang et al. studied DED patients with SS using single-photon emission computed tomography (SPECT). They scanned the salivary glands of the patients with SPECT and calculated different parameters, of which excretion fraction displayed a strong correlation with Schirmer's I test and could be an indicator of lacrimal gland secretion dysfunction.

## Treatment

The therapy of DED has advanced dramatically over the last three decades. With the understanding of DED pathophysiology, a slew of new treatments have emerged, each focusing on a distinct pathway of DED pathogenesis. Liu T. et al. evaluated the effect of punctal plugs in DED patients and analyzed their clinical characteristics before and after surgery. Punctal plugs insertion found to be an effective therapy for DED, specifically aqueous-deficient dry eye, due to increased meibomian gland function and dry eye characteristics. Lin et al. conducted a single-center, randomized, controlled clinical trial to see if hydroxybutyl chitosan (HBC), which is also used for lacrimal drainage system blockage, is effective. HBC is a liquid form plug that, after administration, converts to a hydrogel solid form in 50 s. HBC demonstrated equivalent efficacy to another intracanalicular plug on the market in their trial, and its liquid qualities gave it the advantages of flexibility and individualization, making it a promising technique for DED treatment. Qin et al. inquired into the efficacy of lycium barbarum polysaccharide (LBP), a substance which can suppress inflammation, for the treatment of DED in murine models. They illustrated that topical utilization of LBP performed its anti-inflammatory effects and is capable of improving DED. Liu K. et al. treated meibomian gland dysfunction associated DED patients with a formulation comprised of omega-3 free fatty acids, lutein, aronia extract, vitamin C, and vitamin E. They reported that combining this dietary supplement with traditional DED treatment can further ameliorate indications and symptoms of the patients. Demodex folliculorum infestation, as previously indicated, contributes to the pathogenesis of MGD-related dry eye and could induce chalazion. Relevantly, Zhu, Huang et al. found that treating recurrent chalaziosis with a combination of strong pulse light and meibomian gland expression treatment was effective.

Patients with DED suffer from ocular discomfort, which has a negative impact on their eyesight quality of life. Thus, the correlation between DED and mental illness has gotten a lot of attention. Yu H. et al. enrolled DED patients with anxiety and depression and discussed the relationship between them. They outlined the challenges that DED patients may face through interviews and qualitative research of 47 patients. They also quoted some of the patients to provide a more direct understanding. They pointed out that anxiety and depression may impair a patient's initiative and cooperation, causing the condition to worsen. As a result, in addition to physical and chemical treatments for signs and symptoms of DED, mental health of DED patients should not be disregarded and proper management is necessary.

## Conclusion

Many researches under this topic studied the risk factors of DED that mainly falls into three categories, namely medical conditions, clinical interference and VDT usage. Simultaneously, multiple new mechanisms concerning inflammation and dysbiotic microbiome were revealed in the pathogenesis of DED. These articles illustrated that DED can derive from a wide range of causes through various pathways, highlighting the multifactorial feature of DED. When it comes to diagnosis and treatment, several novel measures that bare promising effects were introduced, which will be of huge benefit to patients of DED. Taken together, this Research Topic has pushed forward our understanding of DED in terms of pathophysiology, diagnosis and treatment. We have great confidence that, in the years to come, the research of DED will see a rapid progress.

## Author Contributions

All authors listed have made a substantial, direct, and intellectual contribution to the work and approved it for publication.

## Funding

JH was supported by the National Natural Science Foundation of China (81970766 and 82171102), the Program for Professor of Special Appointment (Eastern Scholar) at Shanghai Institutions of Higher Learning, the Shanghai Innovation Development Program (2020-RGZN-02033), and the Shanghai Key Clinical Research Program (SHDC2020CR3052B). XZ was supported by the Shanghai Natural Science Foundation (19ZR1408400).

## Conflict of Interest

The authors declare that the research was conducted in the absence of any commercial or financial relationships that could be construed as a potential conflict of interest.

## Publisher's Note

All claims expressed in this article are solely those of the authors and do not necessarily represent those of their affiliated organizations, or those of the publisher, the editors and the reviewers. Any product that may be evaluated in this article, or claim that may be made by its manufacturer, is not guaranteed or endorsed by the publisher.
